# Targeting the androgenic pathway in elderly patients with castration-resistant prostate cancer: A meta-analysis of randomized trials

**DOI:** 10.1097/MD.0000000000004636

**Published:** 2016-10-28

**Authors:** Giandomenico Roviello, Maria Rosa Cappelletti, Laura Zanotti, Angela Gobbi, Chiara Senti, Alberto Bottini, Andrea Ravelli, Alberto Bonetta, Giovanni Paganini, Daniele Generali

**Affiliations:** a Section of Pharmacology and University Center DIFF-Drug Innovation Forward Future, Department of Molecular and Translational Medicine, University of Brescia, Brescia; b U.S. Terapia Molecolare e Farmacogenomica; c Radiotherapy Department, ASST Cremona, Cremona; d Section of Experimental Oncology, Department of Clinical and Experimental Medicine, University of Parma, Parma; e Unit of General Medicine, Azienda Ospedaliera “C. Poma” Presidio Ospedaliero di Pieve di Coriano, Mantova; f Department of Medical, Surgery and Health Sciences, University of Trieste, Trieste, Italy.

**Keywords:** abiraterone acetate, elderly, enzalutamide, orteronel, prostate cancer

## Abstract

**Background:**

The novel hormonal drugs have recently entered in the armamentarium of therapies for treatment of metastatic castration-resistant prostate cancer (CRPC). First reports are available for their use in elderly men with CRPC.

**Method:**

A meta-analysis of randomized controlled trials (RCTs) has been performed. PubMed, the Cochrane Library, and American Society of Clinical Oncology (ASCO) University Meeting were searched for data on the use of new hormonal treatment in elderly patients with CRPC.

**Results:**

Nine studies for a total of 3512 elderly patients were available for meta-analysis. Six studies reported outcomes of patients aged >75 years old while 2 studies reported on patients aged >70 years old. The pooled analysis of the androgen synthesis inhibitors revealed significantly increased overall survival (OS) due to antiandrogen agents compared with placebo or placebo and prednisone (hazard ratio (HR) for death: HR = 0.74, 95% CI: 0.67–0.82; *P* < 0.00001). Moreover, the new antiandrogenic therapy significantly improved the progression-free survival (HR = 0.45, 95% CI: 0.31–0.65; *P* < 0.0001). The incidence of any grade ≥3 adverse effect was only moderately higher during with the antiandrogenic therapy as compared to the control arms (response rate = 1.03, 95% CI: 0.88–1.20; *P* = 0.72).

**Conclusion:**

This study confirmed that agents targeting the androgen axis (i.e., enzalutamide, abiraterone) significantly prolonged OS in elderly men with CRPC.

## Abstract 2

Supplemental Digital Content is available in the text

## 1 Introduction

Prostate cancer can be considered as a disease of the elderly since, it was estimated that the 35% of cases were diagnosed between 65 and 74 years old, and the 25% were diagnosed after 75 years.^[^1 2^]^ These elderly patients are often fragile with several comorbidities, therefore they could have a low tolerability to antitumoral treatments, especially if chemotherapy-based agents. The adequate management of the disease represents also an emerging issue due to the recent availability of new effective agents for the treatment of metastatic castration resistant prostate cancer (CRPC) in either chemotherapy naïve men or chemotherapy refractory,^[3]^ and many patients do not receive optimal therapy as the result of treatment decisions made primarily on the basis of chronological age alone. In this scenario, unfortunately the current guidelines do not make specific recommendations for the treatment of elderly men with prostate cancer. Therefore, there is a clinical need for specific data on elderly patients with CRPC exposed to the new drugs. Abiraterone and enzalutamide are 2 novel agents targeting the androgen axis, both approved for CRPC treatment,^[^4–7^]^ while abiraterone targets a pivotal enzyme for CRPC progression such as the CYP17 enzymes, enzalutamide is a novel potent androgen receptor signaling antagonist. Additionally, orteronel (TAK-700) is a nonsteroidal, reversible, selective 17,20-lyase inhibitor demonstrating some activity in CRPC patients.^[^8 9^]^ First clinical reports suggest these new hormonal agents improved outcomes in both younger and older men, with comparable safety and tolerability.^[^10 11^]^ The nature of these studies, mostly post hoc analyses, does not allow definitive conclusions on the real effects of all these novel hormonal therapies in the elderly CRPC population. Therefore, the aim of this study is to evaluate and analyze the clinical data from randomized controlled clinical trials on the efficacy and safety of new antiandrogenic drugs in elderly patients (over 75 years old) with CRPC.

## 2 Materials and methods

### 2.1 Data retrieval strategies

We conducted this meta-analysis of randomized controlled trials (RCTs) in accordance with the preferences for reported items in systematic reviews and meta-analyses guidelines.^[12]^ PubMed/MEDLINE, the Cochrane Library, and American Society of Clinical Oncology (ASCO) University Meeting were searched for relevant publications using the following terms: “prostate cancer,” “castration-resistant prostate cancer,” “CRPC,” “elderly” and “abiraterone,” “enzalutamide,” “orteronel,” “ARN-509,” “Galeterone,” and “ODM-201.” The publications that were available in these databases up to April 1, 2016, were analyzed. The search was restricted to human studies. The search criteria were limited to phase III or phase II RCTs. The computer search was supplemented with manual searches of the references listed in all of the retrieved review articles, primary studies.

### 2.2 Inclusion criteria

Two independent reviewers screened the studies according to specific selection and exclusion criteria. The inclusion/exclusion decisions regarding contentious studies were made via consultation with a third reviewer. The studies were identified according to the following inclusion criteria: elderly human participants with CRPC; a new antiandrogenic drug interventions; the presence of a control for comparison (placebo or not), a primary outcome of survival expressed as the hazard ratio (HR) and secondary outcomes of progression-free survival (PFS) expressed as HR, time to prostatic antigen specific (PSA) progression expressed as HR, PSA response rate expressed as relative risk (RR), and major adverse effects (any grade 3–4 adverse event) expressed as RR. The following exclusion criteria were used: insufficient data were available to estimate the outcomes; animal studies; the size of each arm was fewer than 10 participants; the presence of a single arm study, and studies conducted before 2010.

### 2.3 Data extraction

Two authors independently extracted the relevant data including the name of the first author, country, publication year, characteristics of the enrolled patients (i.e., age, number, and drug administration), median follow-up, median treatment duration, and information about the study design (i.e., the type of blinding, the type of control, the methods for randomization allocation), survival outcomes expressed as HRs for overall survival (OS) and PFS and time to PSA progression, number of patients who experienced a PSA response of the 50% and any adverse major event (grade 3–4). For each trial, the new hormonal agent ± prednisone was considered to be the experimental arm.

### 2.4 Quality assessment

The methodological quality of each included RCT was assessed by 2 independent researchers (DS and RC). Study quality was assessed using the Jadad 5-item scale, taking into account randomization, double blinding, and withdrawals. The final score ranged from 0 to 5.^[13]^ Disagreements were evaluated by a kappa test and consensus was achieved in discussion with the corresponding author (GR).

### 2.5 Statistical analysis

The statistical analyses were performed with Revman 5.3. The summary estimates were generated using a fixed-effect model (Mantel–Haenszel method) or a random-effect model (DerSimonian–Laird method)^[^14 15^]^ depending on the absence or presence of heterogeneity. Statistical heterogeneity was assessed with the *Q* test and the *I* ^2^ statistic. *I* ^2^ values of 25%, 50%, and 75% were considered to indicate low, moderate, and high heterogeneity, respectively.^[16]^ When *P* > 0.1 and *I* ^2^ < 50%, the fixed-effects model was used; otherwise, the random-effects model was used. For the time-to-event variables, including OS, PFS, time to PSA progression, and time to first skeletal even, the HRs with the 95% confidence intervals (95% CIs) were calculated for each study. For the dichotomous variables, including PSA response and the rate of adverse events, the RRs with the 95% CIs were calculated for each study. Due to the small number of trial that were included, no publication bias was estimated. A subgroup analysis was performed to highlight any differences between studies in pre- and postdocetaxel settings for all end points. For all the statistical analyses, a value of *P* < 0.05 was regarded as statistically significant, and all tests were 2-sided. Ethical approval was obtained from the ethics committee of the University of Brescia.

## 3 Results

### 3.1 Literature review and characteristics of the included studies

**Figure 1:**
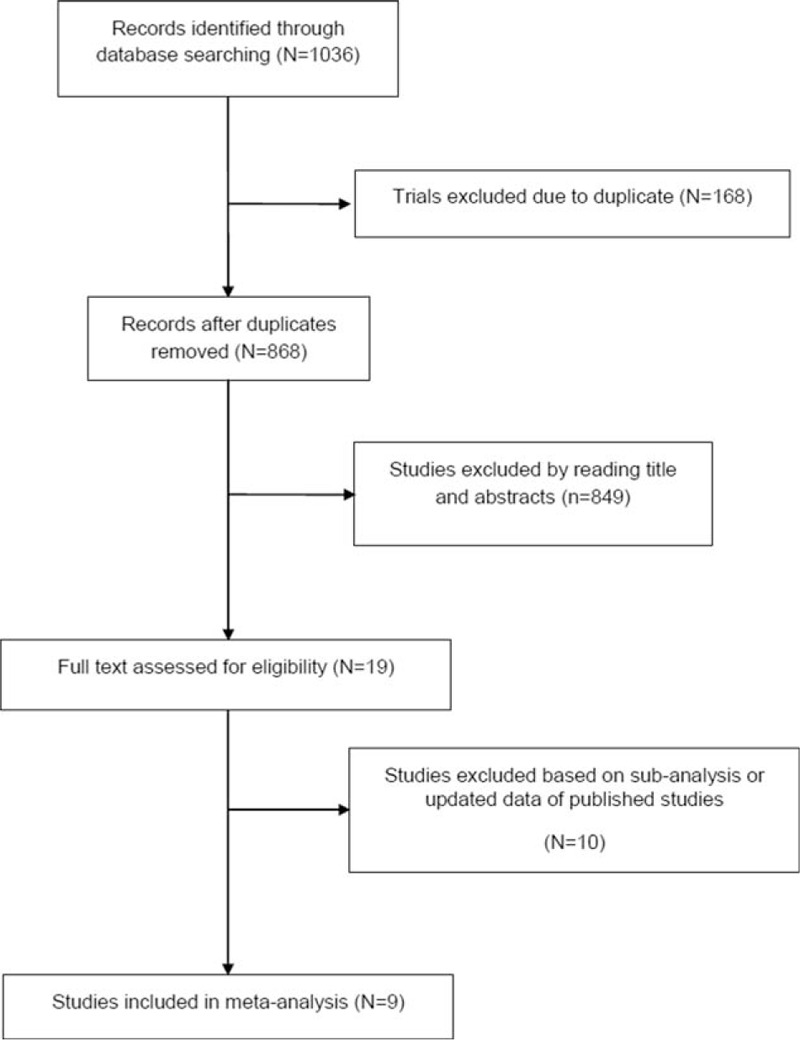
Trial selection flow chart.

Nine studies^[^8–11 17–21^]^ with 1970 cases in the antiandrogenic group and 1542 cases in the control group were included in the meta-analysis according to the inclusion and exclusion criteria described in the Materials and Methods Section (Fig. 1). Six studies involved patients >75 years old,^[^10 11 17–20^]^ while 2 studies^[^8 9^]^ reported data on a subgroup analysis and regarded >70 years old men, one trial reported data on ≥65 years old men.^[21]^ There were 5 postchemotherapy studies that included 1487 cases (917 in the experimental arm and 570 as control arm) and 4 prechemotherapy studies that included 2025 cases (1053 in the experimental arm and 972 as control arm). The patient's characteristics were obtained for all studies. With regard to abiraterone, data has been obtained from 2 post hoc analysis of the COU-AA-301 and COU-AA-302 trial respectively^[^10 18^]^ and a subgroup analysis.^[21]^ With regard to enzalutamide, data have been obtained from a post hoc analysis of the AFFIRM and PREVAIL trials and from a subgroup analysis of TERRAIN and STRIVE trials.^[^11 17 19 20^]^ Finally, for orteronel data were obtained from a subgroup analysis of ELM-PC 4, ELM-PC 5 studies. The characteristics of the studies included in the meta-analysis and their primary end-points are summarized in Table 1. The median Jadad score was 5. All the considered studies were randomized. In 4 studies (COU-AA-301, COU-AA-302, ELM-PC 4, ELM-PC 5, Sun et al) the comparator was placebo plus prednisone, while AFFIRM and PREVAIL compared enzalutamide over placebo, finally in TERRAIN and STRIVE the comparator was bicalutamide.

**Table 1:**
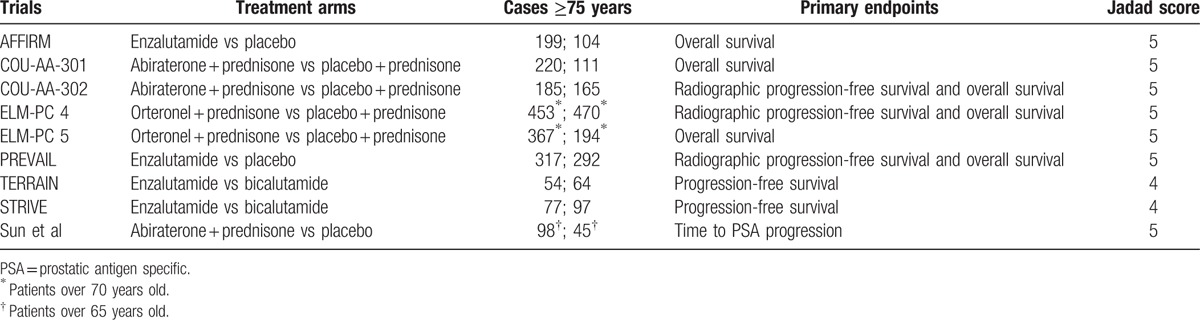
Characteristics of the included studies.

### 3.2 Survival and other end-points

Data about OS and PFS are reported in Table 2. With regard to the mean treatment duration, we observed 11.6 months in the experimental group and 6.5 months in the control group and the AFFIRM trial does not report data on treatment drug duration. An OS higher in the experimental arm has been observed for all the included studies. However, in studies based on the use of orteronel in CRPC, this superiority was not statistically significant.

**Table 2:**
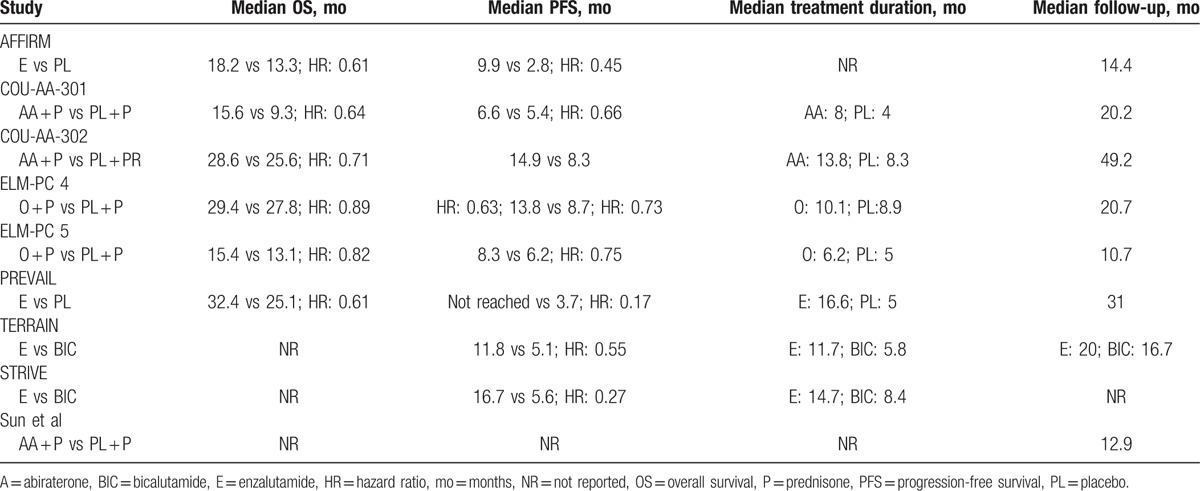
Data on overall survival, median treatment duration, and median follow-up of the included studies.

**Figure 2:**
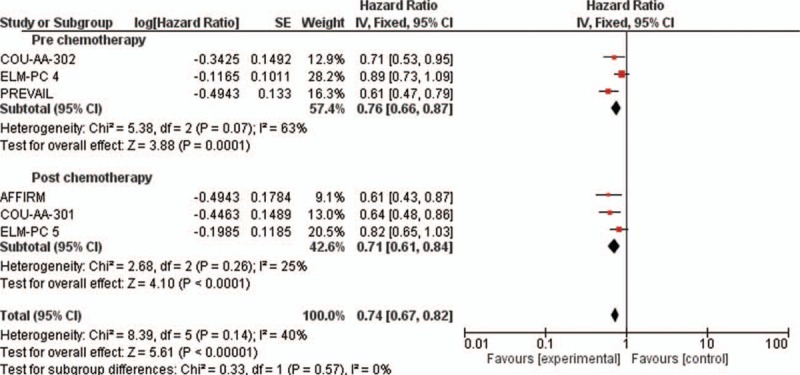
Forest plots of hazard ratios (HRs) for overall survival (OS) comparing new antiandrogenic therapies to control arm. The Chi-squared test showed moderate heterogeneity between the trials. The random effects model was used.

The pooled analysis revealed that the new antiandrogenic therapy significantly improved the OS (HR = 0.74, 95% CI: 0.67–0.82; *P* < 0.00001; Fig. 2) compared with the placebo or placebo with prednisone. The fixed-effects model was used for the analysis of the OSs. In the subgroups analysis of the antiandrogenic combinations used in the pre- and postchemotherapy settings, the results revealed that the therapies with antiandrogenic agents significantly improved the OS to a greater extent in the postchemotherapy setting (HR = 0.71, 95% CI: 0.61–0.84) than in the prechemotherapy setting (HR = 0.76, 95% CI: 0.66–0.87) (Fig. 2). However, the test for evaluating the differences within the subgroup was not statistically significant.

**Figure 3:**
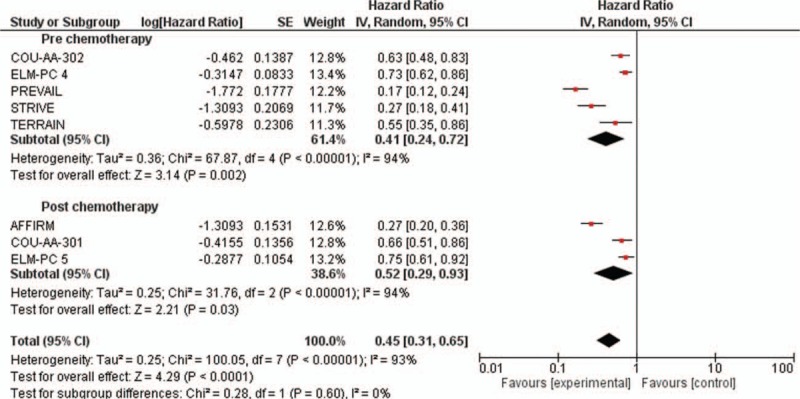
Forest plots of hazard ratios (HRs) for progression-free survival (PFS) comparing new antiandrogenic therapies to control arm. The Chi-squared test showed high heterogeneity between the trials. The random effects model was used.

**Figure 4:**
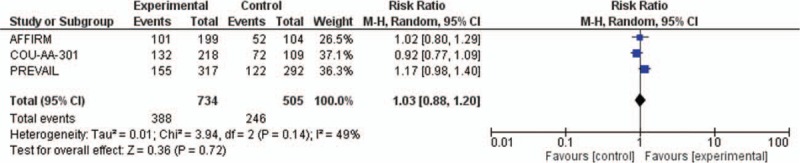
Forest plots of relative risk (RR) for any grade ≥3 adverse effect comparing new antiandrogenic therapies to control arm. The Chi-squared test showed moderate heterogeneity between the trials. The random effects model was used.

Progression-free survival (PFS) was always higher in the experimental arm versus the control arm (Table 2). The pooled analysis revealed the new antiandrogenic therapy significantly improved PFS (HR = 0.45, 95% CI: 0.31–0.65; *P* < 0.0001) (Fig. 3) compared with control (tau^2^ = 0.25). The random-effects model was used for the analysis of the PFSs due to the presence of high heterogeneity (*I* ^2^ = 93%) between the trials. Regarding the other secondary end-points, time to PSA progression and PSA response rate for elderly patients were reported in Supplementary Data Figs. 1 and 2. However, no statistically significant difference has been observed. Finally, adverse events were obtained from COU-AA-301, AFFIRM, and PREVAIL trial. The pooled analysis with a random-effects model also revealed the incidence of any grade ≥3 adverse effect was only moderately higher during with the antiandrogenic therapy (RR = 1.03, 95% CI: 0.88–1.20; *P* = 0.72; tau^2^ = 0.01; Fig. 4) than in the control arms even if this difference was not statistically significant.

## 4 Discussion

The present study is a systematic review and a meta-analysis of RCTs to assess the efficacy and safety of new antiandrogen therapies in elderly patients with CRPC. The new antiandrogens improved the PFS and OS of the elderly patients (mostly >75 years) with CRPC compared with control arm. Therefore, we confirm that targeting the androgenic pathway is efficacious and safe also in the subgroup of elderly CRPC.

Prostate cancer predominantly affects older men with a median age at diagnosis of 68 years and is considered the most prevalent cancer in men over 70 years.^[22]^ Unfortunately, although the role of new antiandrogen therapies in CRPC is well established,^[23]^ the randomized clinical trials usually have strictly inclusion criteria, especially in regard to concomitant disease and comorbidities, limiting the possible enrolment of elderly patients. Moreover, guidelines make no specific recommendations to prostate cancer patient with ages over 70. Noteworthy, older patients are also more likely to present with very advanced disease with a greater risk of death resulting from prostate cancer despite from competing causes.^[2]^ In fact it was shown that, elderly men aged >75 years contributed almost half (48%) of all metastatic cases.^[2]^ In addition, several studies showed the different risk in mortality and nonreceipt of curative treatment for elderly prostate cancer compared to younger. In fact, the Canadian Cancer Registry reported an higher mortality of prostate cancer in older men compared with younger men.^[24]^ Unfortunately, data from a population-based study of 5456 patients have shown that men aged 70 to 79 years had a significant fivefold increased risk of not receiving curative treatment relative to men aged 60 to 69 years.^[25]^ All these facts highlight the importance of data about the use of systemic treatments in elderly CRPC taking also into account the high budget impact of the upcoming novel drugs with diverse mechanisms of action for CRPC.^[26]^

Although, the first reports suggest the efficacy and safety of new antiandrogen therapies in elderly patients with CRPC,^[^10 11 17 18^]^ on the other hand, the last data were derived from a post hoc analysis of clinical randomized trials, therefore, they require caution with further evaluations. In clinical setting, some studies^[^27 28^]^ investigated abiraterone acetate in very elderly patients (octogenarians or ≥85 years aged patients). They have been generated from small, retrospective studies not allowing definitive conclusions. To best of our knowledge, this is the first meta-analysis of more than 3000 patients which support the use of new antiandrogenic therapies in elderly CRPC. In regard to the efficacy end-point, such as OS and PFS, we showed their significant increase due to novel antiandrogen agents compared with placebo, placebo and prednisone and bicalutamide (HR: 0.74 and 0.45, respectively). The success of these novel drugs has reinforced the role of the androgen receptor pathway in the progression of CRPC, highlighting the crucial role of androgens even in patients who have met the criteria of castration resistance. However, the optimum sequence of new agents in CRPC patients is still unclear.^[^29–33^]^ In the near future, more specified trials on the best sequence of treatment are awaited to make definitive conclusions in both elderly and younger patients.

It should underline that elderly men with metastatic CRPC cannot tolerate chemotherapy-induced toxicities such as neutropenia, anemia, and mucositis^[34]^ and to avoid this last adverse event in men aged >75 years, a prophylactic use of G-CSF, especially at cycle 1 could be undertaken. In this contest, our data confirm the good safety profile of novel hormonal agents in CRPC. The pooled analysis with a random-effects model revealed that the incidence of any grade ≥3 adverse effect was only moderately higher during with the antiandrogenic therapy (RR = 1.03). This is an important issue as particular attention should be paid to the use of new agents in the elderly population usually suffering of a number of concomitant diseases. However, more recently several studies showed a relative safety during novel antiandrogenic-based treatments.^[^35–39^]^ First of all, the risk of special adverse events related to CYP-17 inhibitor was never more than 10%.^[35]^ In addition, the long-term treatment with this low-dose corticosteroid that is indispensable to avoid toxicity from abiraterone is safe and tolerable in also elderly patients.^[^36–38^]^ Finally, a recent meta-analysis showed the RR of cardiovascular events in metastatic CRPC patients treated with abiraterone, enzalutamide, or orteronel, is significant increased, however the occurrence of all and grade 3–4 events is very low (about 10%).^[39]^

Nonetheless, this meta-analysis has a few limitations. Data were collected from 4 post hoc analyses; and 4 subgroup analyses of only 7 studies, and these studies exhibited very high levels of heterogeneity for some of the end-points. In addition, the ELM-PC 4 and ELM-PC 5 studies reported data on patients with >70 years of age and not 75 such as the other 4 studies. In the subgroups of the prechemotherapy trials, the studies involved patients who were asymptomatic or mildly symptomatic (COU-AA-302) and only exhibited bone metastases and patients with visceral disease (PREVAIL, ELM-PC 4, and TERRAIN), while the STRIVE trial included also patients without metastatic disease. It should be noted that: the abiraterone and orteronel trials has been comparator to prednisone, whereas placebo were used in the enzalutamide trials except for bicalutamide in TERRAIN and STRIVE; the investigation of the adverse events was limited to number of patients with at least one grade 3–4 event; different versions of CTCAE has been adopted in the trial; specific toxicity data such falls, cognitive impairment, fatigue are lacking; our analysis was based on the literature rather than on individual patients’ data. All these elements could have been affected the definitive results of the analysis.

## 5 Conclusions

In conclusion, our meta-analysis supports the evidence in favor of targeting the androgenic pathway with these novel agents in elderly men with a relative safety profile. In this contest, our analysis which involved patients aged >70 years old and aged >75 years in 4 studies support the use of new hormonal therapies in over 70 years old men.

## Supplementary Material

Supplemental Digital Content
