# Supplementary material for: Targeting the androgenic pathway in elderly patients with castration-resistant prostate cancer: A meta-analysis of randomized trials
Source: Medicine (Baltimore). 2016 Oct 28;95(43):e4636. doi: 10.1097/MD.0000000000004636 (PMC5089083; doi:10.1097/MD.0000000000004636)

**Figure 1.** Analysis of time to PSA progression of the new anti-androgenic therapies compared to control arm in elderly CRPC.


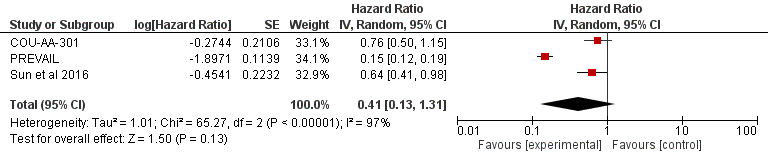


**Figure 2.** Analysis of PSA response of the new anti-androgenic therapies compared to control arm in elderly CRPC.


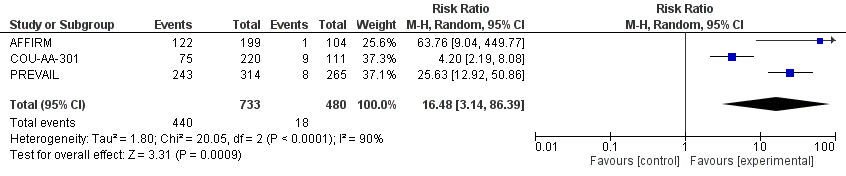

Supplement: Supplemental Digital Content [file medi-95-e4636-s001.docx]
